# Prediction of Physicochemical Properties in Honeys with Portable Near-Infrared (microNIR) Spectroscopy Combined with Multivariate Data Processing

**DOI:** 10.3390/foods10020317

**Published:** 2021-02-03

**Authors:** Olga Escuredo, María Shantal Rodríguez-Flores, Laura Meno, María Carmen Seijo

**Affiliations:** Department of Vegetal Biology and Soil Sciences, Faculty of Sciences, University of Vigo, 32004 Ourense, Spain; mariasharodriguez@uvigo.es (M.S.R.-F.); laura.meno@uvigo.es (L.M.); mcoello@uvigo.es (M.C.S.)

**Keywords:** honey, quality control, authentication, botanical origin, NIR, modified partial least squares, linear discriminant analysis

## Abstract

There is an increase in the consumption of natural foods with healthy benefits such as honey. The physicochemical composition contributes to the particularities of honey that differ depending on the botanical origin. Botanical and geographical declaration protects consumers from possible fraud and ensures the quality of the product. The objective of this study was to develop prediction models using a portable near-Infrared (MicroNIR) Spectroscopy to contribute to authenticate honeys from Northwest Spain. Based on reference physicochemical analyses of honey, prediction equations using principal components analysis and partial least square regression were developed. Statistical descriptors were good for moisture, hydroxymethylfurfural (HMF), color (Pfund, L and b* coordinates of CIELab) and flavonoids (RSQ > 0.75; RPD > 2.0), and acceptable for electrical conductivity (EC), pH and phenols (RSQ > 0.61; RDP > 1.5). Linear discriminant analysis correctly classified the 88.1% of honeys based on physicochemical parameters and botanical origin (heather, chestnut, eucalyptus, blackberry, honeydew, multifloral). Estimation of quality and physicochemical properties of honey with NIR-spectra data and chemometrics proves to be a powerful tool to fulfil quality goals of this bee product. Results supported that the portable spectroscopy devices provided an effective tool for the apicultural sector to rapid in-situ classification and authentication of honey.

## 1. Introduction

Healthy habits are part of our daily life. In recent years, there is a greater concern for health taking care of the daily diet and physical activity. Honey is a healthful food produced by honeybees from floral nectar or secretions of plants or some kind of aphids. Increasing honey consumption can be attributed to the consumer interest in natural foods with health benefits [1,2]. The properties derive from its particular chemical composition, as well as its sensorial characteristics and depend mainly on the flowers and biogeographical regions involved in its production [1,2,3,4,5,6]. Although they are also affected by processing, manipulation, packaging, and storage time [3,4,7], hence ensuring product quality is essential.

The quality and authenticity of its geographical and botanical origins remain as important factors in reliable marketing [2,8]. This bee product can be classified according to their botanical source as unifloral honey (if arising predominantly from a single plant species), multifloral honey (obtained from multiples plant species), or honeydew when was from secretions in plants. In north-western Spain, unifloral honeys of eucalyptus, chestnut, heather, and blackberry are produced, and there is also a good production of honeydew honey [9]. Since 2007, these unifloral honeys in European countries were recognized in the Protected Geographical Indication (PGI) *Miel de Galicia* (Commission Regulation (EC) No 868/2007 of 23 July 2007). Honeydew honeys are currently in revision process to include them in this designation. The increasing demand for unifloral honeys, with protected designation of origin (PDO) and PGI, generally perceived as high-quality products, produced an increase in their commercial value and, at the same time, an increase in counterfeiting [10]. Undoubtedly, food products with high value-added require exhaustive controls examining a large number of samples that guarantee quality [11].

Microscopic pollen analysis is commonly used to determine the botanical origin of honey, and in some cases, the geographical origin [1,12]. Pollen profile is a useful tool in the identification of the main pollen of honey sample, as a result of bees collecting nectar. Accurate identification is aided by standards and pollen databases [13]. Nevertheless, this approach is time-consuming and strongly dependent on the qualifications and experience of the analyst [1,6,13]. Although for exact determination of honey origin, sensory, and physicochemical properties are also needed [12,14,15].

To assess honey quality standard methods are used, including spectrophotometric, refractometric, titration and melissopalynological methods [12,16]. Some quality parameters (such as water content, pH, electrical conductivity, acidity, hydroxymethylfurfural (HMF) content, diastase activity, or reducing sugars) inform about the botanical origin and confirm the adequate manipulation and storage of honey [2,3,16,17].

On the other hand, the color is one of the most common commercial attributes of honey. Consumers have preferences and the particular tonalities in honey depend on the botanical origin, deriving from certain chemical compounds such as the polyphenols, carotenoids, or minerals [14,18,19,20,21]. Consequently, the correct classification of botanical origin of honeys based on color allows beekeepers and exporters to determine the most advantageous market destination for this apicultural product [14].

In the food industry, the concern for public health and detection of possible frauds regarding the labelling of commercial products have led many regulatory bodies to demand rigorous inspections. Hence, raw material identification or verification is a common quality-control practice. However, common analytical chemistry determinations such as high-performance liquid chromatography typically take a long time to complete, is expensive and destructive for samples. Recently, non-destructive characterization tools for quality control based on the principles of spectroscopy were introduced [11]. More specifically, the near-infrared (NIR) technique has expanded its scope of application in the past decade, through the rapid integration of technologies from various fields [7]. Large amounts of spectral data, characterized by an intercorrelation among the recorded spectral variables, can be processed [11]. Unfortunately, this technology is heavily dependent on reference conventional methods to develop a calibration model and to their validation [13,22]. The assessment and interpretation of spectroscopy instrumentation are usually not straightforward, and the application of chemometric approach is crucial to guarantee the success of this technique. Major advantages of NIR spectroscopy are that it requires little to no preparation, its rapidness, safety to the analyst, non-destructiveness, and multicomponent remote analysis [13,23].

NIR technology has been described as a successful tool in analytical instrumentation and quality control in various fields such as food adulteration, authenticity control, the assessment of physicochemical attributes, rheological, or technological properties [1,13,23,24,25,26], as well as in petrochemical industries, plastic contaminants, pharmaceuticals, cosmetics, and medical applications [22,27]. This method has been widely used in the field of honey quality detection, such as in the determination of 5-hydroxymethylfurfural [24,28,29,30], diastase activity [7], moisture and reducing sugar content [23,24,28,31], compounds with antioxidant activity such polyphenols or minerals [1,25], floral origin discrimination [5,6,32], and honey adulteration [33,34].

A high standard of quality for honey is achieved by developing alternative methods that are simple, fast, cheap, and reliable. In this context, the objectives pursued with the present study were (a) assessment the potential of portable microNIR and chemometric techniques for the prediction of main physicochemical parameters in the honey; (b) assessment the ability to discriminate different honey types combining the linear discriminate analysis with main physicochemical attributes and botanical profile. The physicochemical data by conventional methods (moisture, pH, EC, HMF content, diastase index, color, phenols, and flavonoids) of 100 samples from Northwest Spain for the development NIR regression models and discrimination of honey samples were included.

## 2. Materials and Methods

### 2.1. Honey Samples

A total of 100 honey samples of the label Protected Geographical Indication Miel de Galicia (northwest Spain) were used for this study. The samples were collected directly from the beekeepers and stored refrigerated at 4 °C until further analysis. All the physicochemical determinations were conducted in duplicate, after the homogenization of the samples. NIR models were developed using 84 samples for the calibration group and 16 samples for external validation group. Each honey sample were registered by triplicate and the mean value was used for the chemometric treatment.

### 2.2. Palynological Analysis

The pollen analysis of the honey samples was performed based on the method proposed by Louveaux, et al. [35], with some adaptations [9]. The honey samples (10 g) were diluted in distilled water and centrifuged at 4500 rpm for 10 min (first centrifugation), the obtained sediment was re-dissolved and centrifuged for 5 min (second centrifugation). The slide for the microscopical analysis was prepared by duplicate with 100 µL of the sediment. The pollen grains were identified using a Nikon Optiphot II microscope (Nikon UK Ltd., London, UK) at 400× or 1000×. The results were expressed in percentage considering the total number of pollen grains counted.

### 2.3. Determination of Quality Parameters

The moisture was measured with a digital refractometer (ABBE URA-2WAJ-325; Auxilab S.L., Navarra, Spain) on a drop of honey sample. The refractive index values at 20 °C, were converted to moisture content using the Chataway table.

Then, 5 g honey dissolved in 25 mL bi-distilled water were used to determine the pH and the electrical conductivity (EC). The pH was measured directly using a pH meter (Crison micro pH 2001; Crison Instruments S.A., Barcelona, Spain). EC was measured in a honey solution considering the moisture content of sample with a portable conductivity meter (Knick Portamess 913 Conductivity, Beuckestr, Berlin). Results were expressed in µs/cm.

HMF content and the diastase activity were determined following the methodology proposed by Bogdanov et al. [36]. The HMF content (expressed in mg/100 g) was estimated using the White spectrophotometric method. This method considers the difference between the UV absorbance at 284 nm of a honey solution and the same solution after adding bisulphite. The HMF level was calculated after subtraction of the background absorbance at 336 nm using a UV-Visible Spectrophotometer (Jenway 6305 UV-Visible Spectrophotometer, Staffordshire, UK).

The diastase activity was calculated through the hydrolysis rate of the starch solution by the α-amylase present in a honey buffer solution at 40 °C (Schade method). The amount of starch converted was determined measuring the absorbance of the honey solution at 660 nm for different time points until an endpoint when the absorbance was less than 0.235. An UV-Visible spectrophotometer (Jenway 6305 UV-Visible Spectrophotometer, Staffordshire, UK) was used for this purpose. Diastase activity was expressed as diastase number (DN) or grams of starch hydrolyzed each hour per 100 g honey at 40 °C.

### 2.4. Determination of Color

The color of samples was determined with a portable Minolta Chroma Meter CR-210 colorimeter (Konica Minolta, Tokyo, Japan). The CIE L a*b* coordinates were registered, where L is the luminance component (ranging from 0 to 100), while a* and b* are chromatic coordinates related with the gradient red/green and yellow/blue, respectively.

A HANNA Honey Color C221 colorimeter was also used to determine the color in mm Pfund. The honey must be fluid to be analyzed correctly. For crystallized or weakly fluid honeys, they were heated up to 45 °C in a thermostatic bath until the complete fluidification. Approximately 4 mL of sample was placed in a cuvette and sonicated to eliminate bubbles. The measure was taken after calibration with glycerin (Glycerol HANNA instruments).

### 2.5. Determination of Total Phenol and Flavonoid Content

Folin–Ciocalteu spectrophotometric method adapted to honey was used for the determination of the phenol content [37]. This method is based on the oxidation of the phenolic compounds forming a bluish complex. The samples (0.1 g/mL) were mixed with the reactive of Folin–Ciocalteu and the absorbance at 765 nm was measured using a UV–Vis spectrophotometer (Jenway 6305, UK). Gallic acid solutions (0.01–0.50 mg/mL) as a reference standard were used. Finally, the total phenolic content was expressed as gallic acid equivalents in mg/100 g honey.

The total flavonoid content was measured by spectrophotometry using the Dowd method adapted by Arvouet-Grand et al. [38]. The method uses a solution of aluminum chloride that reacts with the flavonoids present in the honey solution (0.33 g/mL). Solutions developed a yellow color for which absorbance was determined spectrophotometrically at 425 nm. Quercetin solutions (0.002–0.01 mg/mL) as reference standard were used. The results were expressed as equivalents of quercetin in mg/100 g honey.

### 2.6. Near Infrared Spectroscopy Measurement

**Figure 1:**
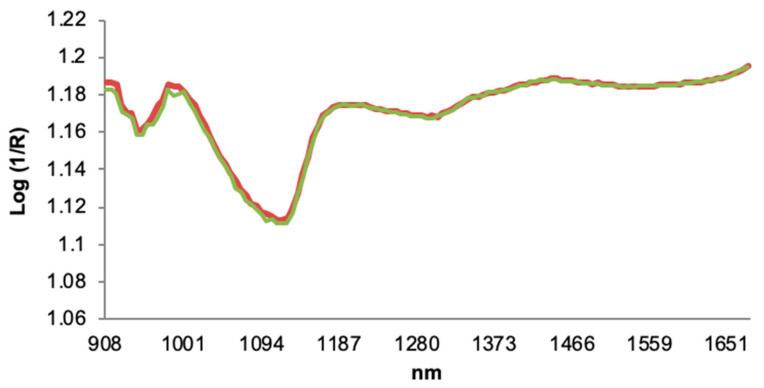
Near-Infrared (NIR) spectra of honey samples recorded by portable MicroNIR.

A portable MicroNIR Pro v2.5 equipment (MicroNIR 1700 ES, VIAVI, Santa Rosa, CA, USA) was used for acquisitions of NIR spectra data on honey samples (Figure 1). The samples properly homogenized were placed to the spectrometer housed in 4 mm borosilicate glass vials with measurements performed through the bottom of the vials. The vials were coupled directly with an adapter to the system NIR, and each sample a minimum of three times was scanned. The NIR spectrophotometer includes an instrument designed to measure diffuse reflectance in the NIR region of the electromagnetic spectrum [39]. A tungsten light bulb composes the system as the radiation source, and a linear-variable filter (LVF) connected to a linear indium gallium arsenide (InGaAs array detector) are integrated into the equipment itself. This equipment used a Spectralon^®^ ceramic tile as a white reference (100% reflectance) of politetrafluoroetilen (~99%). Therefore, a 99% diffuse reflectance panel was used for the 100% reference value, and the 0% reference value was taken by leaving the tungsten Lampson with an empty support (known as dark Current Scan). Spectra were recorded using the instrument acquisition software MicroNIR™ Pro v.2.2 (VIAVI, Santa Rosa, CA, USA). The measuring of reflectance in the NIR zone was recorded in a range between 900 and 1700 nm at intervals of 6 nm in the spectra. To minimize sampling errors, all the samples were analyzed in triplicate and the mean was used in statistical treatments. The diffuse reflectance signal of the NIR spectrum is referred to as reflectance (R), using log (1/R) values for performing chemometric analyses [40]. The advantage of portable MicroNIR systems is its easy handling and size. The MicroNIR dimensions are 45 mm in diameter and 42 mm in height, weighing about 60 g, and it is equipped with a 128-pixel detector array. In addition, the MicroNIR can be directly connected to a USB port of any laptop [27].

### 2.7. NIR-Chemometric Analyses

Chemometric techniques for the calibration of the main physicochemical parameters of honey were applied. Spectral data of samples corresponding to the samples of the calibration set were analyzed by principal component analysis (PCA) [41]. Anomalous spectra were detected by applying the Mahalanobis distance (H-statistic). Considering an H-value greater than 3 (the spectra not belonging to the population), the equations are not used to make any prediction. The modified partial least squares (MPLS) regression method was used to obtain the NIR equations. Partial least squares (PLS) regression is similar to principal component regression (PCR) but uses both reference data and spectral information to form the factors useful for fitting purposes. Using the T ≥ 2.5 criterion, samples that presented high residual values when they were predicted were eliminated from the set. Therefore, statistical parameters of the calibration were obtained for each of the components after removing the samples for spectral (H criterion) or chemical (T criterion) reasons. To optimize the multivariate regression equations, the spectral scattering effects were taken into account with several mathematical treatments: multiplicative scatter correction (MSC), standard normal variate (SNV), D-trend (DT), and SNV-DT [42]. A nomenclature using 4 digits was used (1,4,4,1), in which the first digit is the number of the derivate, the second is the gap over which the derivative is calculated, the third is the number of data points in a running average or smoothing, and the fourth is the second smoothing.

Cross-validation is recommended to select the optimal number of factors and to avoid over fitting [43]. The calibration set is divided into several groups for the cross-validation. Each group is then validated using a calibration developed on the other group of samples. Validation errors generated are combined into a root mean square error of cross-validation (RMSECV). This statistic is considered the best single estimate for the prediction capability of the equations [44]. Cross-validation was performed by splitting the population into eight groups for all cases.

The performance of the models was determining by the squared correlation coefficient for predicted versus measured quantified in cross-validation and the ratio of standard deviation (SD) to SECV of the data set. RPD (ratio of performance to deviation) is the relation between SD and RMSEC, and it is desired to be larger than 2 for a good calibration, and an RPD ratio less than 1.5 indicates poor predictions and the model cannot be used for further prediction [44]. The statistics used to select the best equation for each physicochemical parameter were the highest RSQ (multiple correlation coefficients) and the lowest SECV (standard error of cross-validation) [25]. The software used for chemometric analysis was WinISI II version 1.50 (Infrasoft International, LLC, Silver Spring, Maryland, MD, USA).

### 2.8. Linear Discriminant Analysis

Linear discriminant analysis (LDA) is a supervised classification technique, which uses a class member known for the analysis. In this case, the known variable was honey type determined by palynological analysis. Considering the pollen profile, six honey groups were characterized: heather, chestnut, eucalyptus, blackberry, honeydew, and multifloral. LDA was applied to the collected reference data set (physicochemical and botanical data) to determine a linear combination of these groups of subjects. LDA is considered as a dimensional reduction method to determine a lower dimension hyperplane on which the points will be projected from the higher dimension space [10]. A linear function of the variables is sought which maximizes the ratio of between-class variance and minimizes the ratio of within-class variance. STATGRAPHICS Centurion XVI software (Statpoint Technologies, Inc., The Plains, VA, USA) was used for treatment of data.

## 3. Results

### 3.1. Physicochemical Properties of Honeys: Reference Values

The descriptive analysis of physicochemical data obtained by reference methods are summarized in (Table 1). The data are expressed in function of two groups established for the NIR treatment (calibration and validation).

### 3.2. NIR Calibration Equations

The calibration process was performed with chemometric techniques using the spectra and the physicochemical data of the samples. The samples were split randomly in two groups: calibration group with 84 samples, and external validation group with 16 samples (Table 1). Firstly, a principal component analysis was carried out with the samples corresponding to the calibration group. The spectral variability explained ranged between 99.36% and 99.99%. The principal components required for each parameter were 8 for moisture, EC, HMF and L and b* coordinates, phenols and flavonoids; 5 for pH; 7 for diastase index; 9 for a* coordinate; and 6 for color (Pfund scale). During the cross-validation process, the identified outliers with the T and H criteria were removed of the calibration set. before of the development of equations. According to both criteria were deleted the following samples: 13 for moisture, 10 for EC, eight for pH, 29 for HMF, 11 for diastase index, 11 for Pfund, 15 for L, a* and b* coordinates, 11 for phenols, and 13 for flavonoids.

Calibrations were performed by MPLS using spectral data and the physicochemical data of honey. Table 2 shows the best mathematical treatment, the concentration range, standard deviations and the calibration descriptors for each parameter. The obtained results indicated that it was possible to predict most of the physicochemical parameters in honey samples with portable microNIR system. The degree to which the calibration fits the data set was calculated by considering the highest RSQ, and the lowest SEC and SECV. Moisture, EC, HMF, Pfund, L and b* coordinates of CIELab scale, phenols, and flavonoids presented high RSQ coefficient (between 0.74 and 0.90). Values of RSQ lower than 0.70 had pH, diastase index and a* coordinate of CIELab. Although, the standard errors of calibration (SEC) and of cross-validation (SECV) were acceptable in all cases, presenting a minimum difference, which is an indicator that the NIR models obtained are suitable in the ranges indicated with the portable microNIR.

#### 3.2.1. Internal Validation of Models

**Figure 2:**
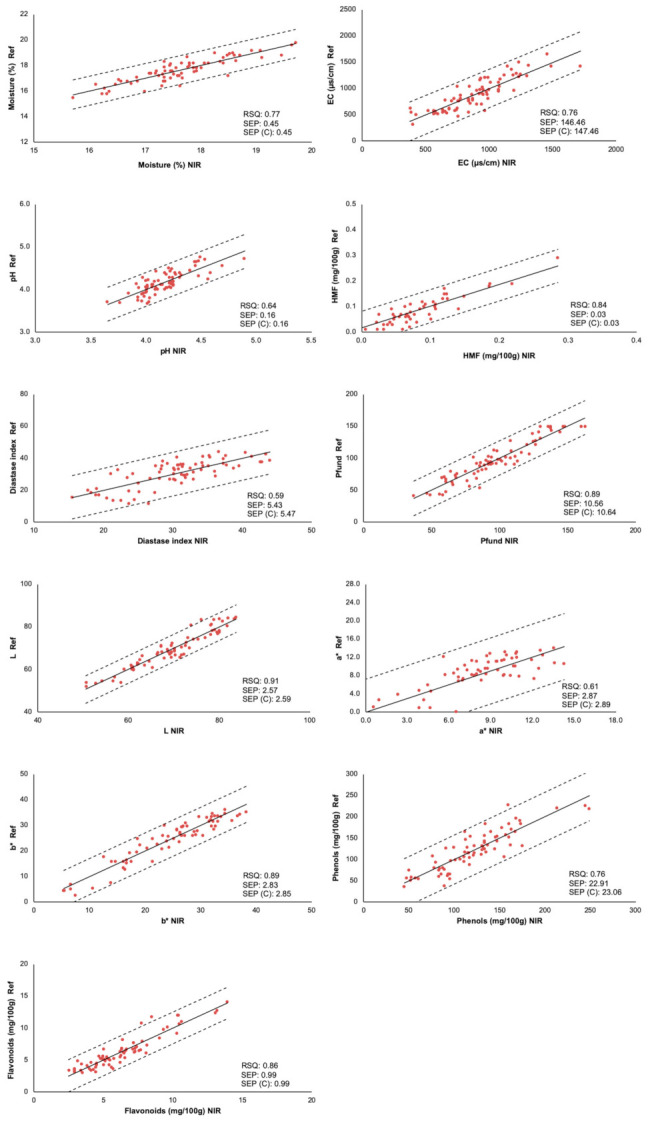
Measured references values versus the NIR values in the prediction set of honey samples.

Cross-validation method (internal validation) was used to study the predictive capacity of the obtained models. The samples of the calibration set were divided into a series of subsets. Six cross-validation sets in all cases were checked, one group for the results (prediction) and the other to construct the calibration model. The process was implemented as many times as there were groups, such that all of them passed through the calibration set and the prediction set. The results of internal validation (predicted values versus reference) for physicochemical variables in NIR is shown in (Figure 2). Considering the statistics SEP and SEP corrected (C) was deduced that the calibration models for moisture, EC, HMF, Pfund, a* and b* coordinates, phenols, and flavonoids were adequate. Therefore, the estimation of these physicochemical parameters was possible with good results.

The RPD statistic was used to determine the predictive capacity of reference methods for NIR calibration. The parameters of moisture, HMF, Pfund, a* and b*, and flavonoids had the higher values of RPD (>2.0). This is indicative of a good calibration of the data. While, EC, pH, and phenols showed an acceptable calibration, with values of RPD higher than 1.5. Diastase index and L coordinate had a RPD value of 1.5, resulting the poorest model.

#### 3.2.2. External Validation of the Models

In external validation the solidity of the method is checked with 16 new honey samples which were not used in the calibration models (Table 1). The average of the sample spectra was taken, the equations obtained were applied, and the NIR values were compared with the reference in accordance with the residuals and the root mean square error (RMSE). In general, the results obtained for each physicochemical parameter analysed were satisfactory. The means of the residuals were between 0.12 for HMF and 188.37 for EC. RMSE values were between 0.18 and 270.36 for HMF and EC, respectively. The predicted values by the calibration models were compared with the reference data using the Student test for paired values (*p* < 0.05). The significance level showed that there were no differences between the results obtained (values were higher than 0.05), ranging the value between 0.07 for L coordinate and 0.92 for moisture (Table 3). Therefore, it can be concluded that the method provides significantly comparable data to the reference physicochemical data.

### 3.3. Botanical Origin of Samples: Pollen Fingerprint

Eighty-four pollen types corresponding to 50 botanical families were identified in the honey samples. The best represented families were Fagaceae, Leguminosae, Ericaceae, Rosaceae, and Myrtaceae. According to their botanical origin, 10 samples were classified as blackberry honey (*Rubus*), 22 samples as chestnut honey (*Castanea sativa*), 9 as eucalyptus honey (*Eucalyptus*), 5 as heather honey (*Erica*), 18 as honeydew honey, and 36 as multifloral honey. The principal pollen types in each honey type are showed in (Table 4).

The blackberry honeys had a mean value of 58.5% for *Rubus* pollen, while *Castanea* was secondary pollen (26.4%). Other important pollen types in sample were *Erica*, *Cytisus* type, *Eucalyptus*, *Echium,* and *Trifolium* type. The averaged percentage of *Castanea* in chestnut honeys was 76.1%. *Rubus* was also present in all samples with a mean value of 14.2%. Other important pollen types were *Cytisus* type, *Erica*, *Trifolium* type, and *Echium*. For eucalyptus honeys, *Eucalyptus* pollen had a mean value of 72.8%. Other significant pollen types were *Cytisus* type, *Castanea*, *Rubus*, *Erica*, *Conium maculatum* type, and *Salix.* In heather honeys, *Castanea* pollen was the pollen type with the highest mean value (37.9%) and a representation of 100% of the samples and the mean value for *Erica* was 35.5% corresponding to a slightly underrepresented pollen in samples. *Cytisus* type, *Eucalyptus*, and *Rubus* were also present in all samples.

The predominant pollen type in honeydew honey was *Castanea*, commonly found as dominant pollen (mean value of 52.3%). *Rubus* is usually secondary pollen and *Cytisus* type, *Erica*, *Plantago*, and *Salix* were also well represented pollen.

Multifloral honeys had diverse pollen spectra. Commonly *Castanea* was the main pollen type and *Eucalyptus* secondary pollen while, *Rubus*, *Cytisus* type, *Erica*, *Trifolium* type, *Salix*, *Echium*, and *Cynoglossum* appeared as other significant pollen.

### 3.4. Discrimination of the Samples by Honey Type

LDA was used to classify honey samples according the honey type. Main pollen types (*Castanea sativa*, *Eucalyptus*, *Erica*, and *Rubus*) and physicochemical characteristics (moisture, EC, pH, HMF, diastase index, color, phenols, and flavonoids) were included.

Five discriminant functions represented the 100% of variability of the data in the discriminant analysis (Table 5). The cumulative contribution rate of the first two linear discriminant functions accounted for 66.07%, which represented the largest fraction of overall variability in the dataset. In the first function a Wilks’ Lambda = 0.01, Chi-Square = 432.3, DF = 75, *p* < 0.01 was formed; and in the second function a Wilks’ Lambda = 0.04, Chi-Square = 296.4, DF = 56, *p* < 0.01. The significant value (*p* < 0.05) of Wilks’ Lambda showed that the discriminant function was basic for the differentiation of the investigated groups. In addition, the higher values of eigenvalue and canonical correlations showed the high power of discrimination of the first two functions.

**Figure 3:**
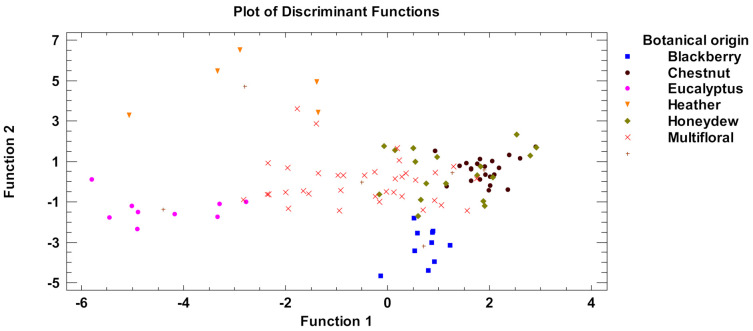
Plot of the first two functions of linear discriminant analysis based on main pollen types and physicochemical characteristics of honeys.

Figure 3 shows the representation of the first two functions of discriminant analysis applied to all the honey samples. The graphical projection shown that honey types were satisfactorily differentiated. The overall correct classification rate was 88.1% for all samples (Table 6). The groups of unifloral samples (heather, eucalyptus, and blackberry) showed a correct classification (100% of samples) with the higher discrimination rate. Chestnut honeys and honeydew honeys were correctly classified with 83.4% and 83.3%, respectively. Finally, multifloral honeys had a correct classification of 83.3%. Regarding chestnut and honeydew honeys, 3 and 2 samples, respectively, were interchanged of group. This is possible due to the closeness of these samples, produced in same biogeographical area and from the same plant species but with different predominance. For this reason, some samples are difficult to classify being possible they are chestnut honeys with honeydew contributions.

## 4. Discussion

The increasing consumption of natural products demands authenticity evaluations [33]. The first step in authenticity is the knowledge of a common pattern in samples with the same botanical and geographical origin. This is increasingly demanded by beekeepers, consumers, and by control entities. Furthermore, declarations of the botanical and geographical origin of honey are important to protect consumers from possible frauds and quality flaws.

In this context, the development of rapid and easy techniques to check quality and to typify many samples in a short time is a challenge. Some analytical methodologies aimed at foods authentication and traceability have been developed [11]. The best known is the NIR technology, which is enjoying increasing popularity in the fresh food and food processing industry. NIR spectroscopy represents an emerging analytical technique due to its low running costs, simple, non-destructive, environmentally friendly, rapid application, and allows several analytes to be detected simultaneously in large number of samples [7,23,26].

Quantitative or qualitative chemometric tools are necessary for the development of calibration or classification methods of groups of samples. The calibration of NIR models allows estimating with accurately desired parameters in unknown samples, but with a previous calibration with reference data obtained by conventional methods. PLS is applied in order to extract analytical information from the spectra and it has contributed much to spectral analysis works [7,26,40]. This method was used to develop quantitative calibration models on honeys matrix [1,7,23,24,25,29]. The correlation of the NIR spectral information with the main physicochemical parameters of reference in honey was analysed in the present study in order to assess the usefulness of portable microNIR in the analysis of quality and authenticity of this bee product. The use of this portable instrument constitutes an opportunity to honey industries to follow the progress of a product throughout the manufacturing process (traceability), to ensure food security movement, and to take trade decisions. The best calibration models were obtained for HMF, color (Pfund, L and b* coordinates by CieLAB) and flavonoid content, with a high capacity of prediction (RPD > 2). However, moisture, EC, pH, and phenols had lower values of RPD but acceptable (RPD > 1.5).

HMF and diastase content in honey are important parameters for assessing the quality and particularly its freshness. According to the Honey Quality and International Regulatory Standards, HMF must be absent or present in very low concentrations (with a maximum limit of 40 mg/kg), and the diastase activity must not be less than or equal to eight in fresh honey. All the honey samples analyzed in this study met these reference standards. Apriceno et al. [29] showed excellent results in Italian honeys based in NIR spectroscopy, with correlation coefficient of prediction of 0.98 and RPD higher than three. However, the prediction accuracy for HMF in honey by NIR was poor and unreliable in other studies [24,28]. Perhaps this is due good results are obtained with a large number of samples and it is sometimes difficult to work with quality samples knowing the provenance. Stöbener et al. [30] developed models for determining HMF content in honey using by Fourier transform attenuated total reflection infrared spectroscopy (ATR-FTIR), resulting coefficients of determination for calibration higher than 0.8. However, they observed for the proper development of a method used to determine trace compounds (such is the case of HMF), it is necessary to use a large number of calibration samples in a wide range of concentrations. Diastase is one the most important enzymes of honey, enriches the nutritional and therapeutic function of this bee product, and it is used as an important index to evaluate honey qualities [7]. In addition, the traditional chemical method for the determination of diastase content is complicated and time-consuming. Therefore, there is an urgent demand for a non-destructive and rapid method to measure this enzyme. However, the predictive capacity by NIR with the samples set selected in the present study for diastase activity was not satisfactory. Huang et al. [8] showed for diastase activity a prediction coefficient of 0.89 through a heating process based on visible and near-infrared spectroscopy. These researchers, in addition to the dependence of temperature and heating time on the diastase content, confirmed the relationship of the enzyme with the botanical origin of honey.

The range of estimation of phenols and flavonoids was similar to reported by a NIR benchtop equipment [25]. However, the results obtained in the calibration by PLS were better (with RSQ > 0.89 and RPD > 3). The polyphenols are secondary plant metabolites, which are determinants in the sensory and nutritional quality of honey, and they are recognized as having high scientific and therapeutic interest. The association of color with botanical origin and its important role in characterization of honey is well reported [9,14,19,21]. The highest polyphenolic content related with dark honeys (heather, chestnut or honeydew honeys), manifesting the importance of this parameter in the authenticity of honeys. According to Sipos et al. [13] the objective characterization of the color of different products and quantification of the differences between them is a fundamental area of research. Food color is the first sensation that consumers perceive, and it greatly influences their decision to purchase [45]. It is therefore important to distinguish small nuances in color that can differentiate similar honey as dark shades, as occurred with heather, chestnut and honeydew honeys. Therefore, the determination of different physicochemical characteristics combined with statistical techniques is essential to distinguish common patterns in samples.

As we have commented previously, different physicochemical properties have been used to authenticate the botanical origins of honey worldwide. Among the physicochemical parameters best differed the botanical origin of honeys were pH, EC, color, sugar profile, phenols, flavonoids, minerals, and volatile profile [2,3,4,14,16,17]. Among chemometry applications, LDA is used to differentiate groups of samples based on a common pattern. LDA confirmed that honeys of heather, eucalyptus and blackberry analysed in present study were 100% correctly classified. In the case of chestnut and honeydew honeys, the great similarity in some physicochemical characteristics complicates their differentiation, although the classification in this study resulted satisfactory (>83%). The high discrimination power of conventional physicochemical and color parameters by LDA has been previously reported for unifloral honeys. The variables with the greatest discriminatory power using LDA for unifloral honeys (*Citrus* and *Eucalyptus*) from southern Spain were water activity and EC [46]. *Citrus* honeys from Greece, Egypt, Morocco, and Spain correctly classified with rates of 97.3% [8]. Recently, combining physicochemical and botanical variables with LDA, correctly discriminated (97.6%) chestnut and honeydew honeys produced in the Northwest Spain [9].

Honey is the most researched bee product. However, some of the common determinations (such as those referring to quality parameters), beekeepers require qualified laboratories to guarantee the quality of the products in market. This implies more time for the commercialization of honey and an economic cost. Undoubtedly, the development of NIR technology with spectral-chemical information, as well as the application of portable equipment such as microNIR is an alternative for the beekeeping sector. The results obtained shows that portable microNIR equipment is a useful alternative that is comparable to the conventional technology for the determination of main physicochemical parameters in honey. The demand for increasingly versatile equipment by the food industry means that innovative and technological development is present. Development of portable vis/NIR systems including linear variable filter (LVF) of low-cost, with innovation in optical system design, miniaturization of equipment, applying it to non-professionals, which communicates through wireless technology [7,22] make them more attractive. The possibility of in situ identification of the quality parameters and physicochemical properties of honey by beekeepers in industries without destruction of sample is a desired advantage.

## 5. Conclusions

Versatility is a well-defined quality of portable NIR equipment and meets the needs of the primary sector in search of fast and competitive solutions. Results presented in this study showed that NIR spectroscopy by portable microNIR might be useful for the identification of floral origin of honeys from Northwest Spain. Although further experiments are proposed to build a robust database, which could support the use of this equipment as a quick alternative for honey authentication. Specifically, for HMF and diastase index, it would be interesting to establish temperature thresholds that allow adjusting the prediction with more reliable results in terms of its application.

## Figures and Tables

**Table 1:** Reference physicochemical data of honey samples selected for the calibration and validation sets.

	Calibration Set (*N* = 84)				Validation Set (*N* = 16)			
	Mean	SD	Min	Max	Mean	SD	Min	Max
Moisture (%)	17.9	1.1	15.5	20.6	18.0	1.0	16.2	19.6
EC (µs/cm)	869.2	307.6	302.5	1649.5	825.5	296.8	277.0	1334.5
pH	4.2	0.3	3.7	4.8	4.2	0.3	3.7	4.7
HMF (mg/100 g)	0.1	0.2	0.0	1.7	0.1	0.2	0.0	0.5
Diastase index	29.0	9.3	10.1	44.0	27.9	8.0	13.6	36.7
Pfund (mm)	98.2	31.2	41.0	150.0	91.0	35.5	36.0	150.0
L	69.5	10.4	51.9	84.5	74.5	16.7	53.2	88.0
a*	7.4	5.2	−3.1	14.6	4.8	8.3	−3.9	14.2
b*	23.1	9.2	2.8	36.2	22.4	10.7	2.3	33.5
Phenols (mg/100 g)	126.3	51.9	36.3	254.5	120.3	63.8	24.6	233.3
Flavonoids (mg/100 g)	6.7	3.2	3.0	16.7	6.1	3.3	2.8	15.7

EC: electrical conductivity; HMF: hydroxymethylfurfural; N: number of samples; SD: standard deviation.

**Table 2:** Statistical descriptors of modified partial least squares (MPL)S calibration models for each physicochemical parameter.

Variable	Math Treatment	N	Mean	SD	Est. Min	Est. Max	SEC	RSQ	SECV	RPD
Moisture (%)	None 1,4,4,1	71	17.6	1.0	14.8	20.5	0.5	0.75	0.8	2.0
EC (µs/cm)	None 1,4,4,1	74	891.2	301.5	0.0	1795.8	155.1	0.74	217.9	1.9
pH	None 0,0,1,1	76	4.2	0.3	3.4	5.0	0.2	0.61	0.2	1.6
HMF (mg/100 g)	None 1,4,4,1	55	0.1	0.1	0.0	0.3	0.0	0.83	0.1	2.4
Diastase index	Detrend only 1,4,4,1	73	30.3	8.6	4.6	56.1	5.8	0.54	8.0	1.5
Pfund (mm)	None 2,10,10,1	73	96.9	31.6	2.0	191.8	11.3	0.87	20.3	2.8
L	Detrend only 1,4,4,1	69	69.3	8.7	43.2	95.3	2.8	0.90	5.4	3.2
a*	None 2,4,4,1	69	7.5	4.6	0.0	21.4	3.0	0.57	3.9	1.5
b*	None 1,4,4,1	69	24.2	8.8	0.0	50.6	3.0	0.88	5.6	2.9
Phenols (mg/100 g)	Detrend only 2,8,6,1	73	120.2	47.3	0.0	262.2	24.5	0.73	38.7	1.9
Flavonoids (mg/100 g)	None 2,8,6,1	71	6.4	2.7	0.0	14.4	1.1	0.84	2.1	2.5

EC: electrical conductivity; HMF: hydroxymethylfurfural; N: number of samples; SD: standard deviation; RSQ: multiple correlation coefficients; SEC: standard error of calibration; SECV: standard error of cross-validation; RPD: ratio performance deviation.

**Table 3:** External validation of physicochemical properties in honeys by NIR (16 samples).

Constituent	Mean Residual	RMSE	*p*
Moisture (%)	0.87	1.02	0.92
EC (µs/cm)	188.37	270.36	0.16
pH	0.25	0.28	0.33
HMF (mg/100 g)	0.12	0.18	0.26
Diastase index	9.25	10.82	0.12
Pfund (mm)	24.81	29.12	0.18
L	10.21	14.76	0.07
a*	6.76	8.65	0.20
b*	6.09	7.68	0.58
Phenols (mg/100 g)	43.11	52.51	0.55
Flavonoids (mg/100 g)	1.92	2.58	0.83

EC: electrical conductivity; HMF: hydroxymethylfurfural; RMSE: root mean standard error.

**Table 4:** Main pollen types identified in the samples by type honey.

Honey Type	N	Main Pollen Type	Secondary Pollen Types	Other Significant Pollen Types
Blackberry	10	*Rubus* (58.5%)	*Castanea* (26.4%)	*Erica*, *Cytisus* type, *Eucalyptus* *Echium*, *Trifolium* type
Chestnut	22	*Castanea* (76.1%)		*Rubus*, *Cytisus* type, *Erica*, *Trifolium* type, *Echium*
Eucalyptus	9	*Eucalyptus* (72.8%)		*Cytisus* type, *Castanea*, *Erica*, *Rubus*, *Conium maculatum* type, *Salix*
Heather	5	*Erica* (35.5%)	*Castanea* (37.9%)	*Cytisus* type, *Eucalyptus*, *Rubus*, *Echium*, *Plantago*
Honeydew	18	*Castanea* (52.3%)	*Rubus* (18.6%)	*Cytisus* type, *Plantago*, *Salix*, *Erica*
Multifloral	36	*Castanea* (41.3%)	*Eucalyptus* (21.0%)	*Rubus*, *Cytisus* type, *Erica*, *Trifolium* type, *Salix*, *Echium* *Cynoglossum*

Main pollen type: predominant pollen type in samples (frequently dominant pollen but not always). Secondary pollen types: 15–45% of pollen spectra; Other significant pollen types: <15% of pollen spectra.

**Table 5:** Discriminant functions and statistics extracted of linear discriminant analysis.

Discriminant Function	Eigenvalue	Relative Percentage	Canonical Correlation	Wilks Lambda	Chi-Square	DF	*p*
1	3.64	38.77	0.89	0.01	432.27	75	<0.001
2	2.56	27.30	0.85	0.04	296.44	56	<0.001
3	1.73	18.37	0.80	0.13	183.97	39	<0.001
4	0.98	10.44	0.70	0.34	95.25	24	<0.001
5	0.48	5.13	0.57	0.67	34.79	11	<0.001

*p*: significance level.

**Table 6:** Results of classification of honey samples considering the linear discriminant analysis.

	Groups		Predicted Group Member					
Honey Type	N		Heather	Chestnut	Eucalyptus	Blackberry	Honeydew	Multifloral
Heather	5	N	5	0	0	0	0	0
		%	100	0	0	0	0	0
Chestnut	22	N	0	19	0	0	3	0
		%	0	83.4	0	0	13.6	0
Eucalyptus	9	N	0	0	9	0	0	0
		%	0	0	100	0	0	0
Blackberry	10	N	0	0	0	10	0	0
		%	0	0	0	100	0	0
Honeydew	18	N	0	2	0	0	15	1
		%	0	11.1	0	0	83.3	5.6
Multifloral	36	N	2	1	1	1	1	30
		%	5.6	2.8	2.8	2.8	2.8	83.3
Percent of cases correctly classified: 88.1%								

N: number of samples; %: correct classification.

## Data Availability

Data is contained within the article.
